# Association Between Different DVT Prevention Methods and Outcomes of Septic Shock Caused by Intestinal Perforation in China: A Cross-Sectional Study

**DOI:** 10.3389/fmed.2022.878075

**Published:** 2022-04-27

**Authors:** Lu Wang, Xudong Ma, Huaiwu He, Longxiang Su, Yanhong Guo, Guangliang Shan, Ye Wang, Xiang Zhou, Dawei Liu, Yun Long

**Affiliations:** ^1^State Key Laboratory of Complex Severe and Rare Diseases, Department of Critical Care Medicine, Peking Union Medical College Hospital, Peking Union Medical College and Chinese Academy of Medical Sciences, Beijing, China; ^2^Department of Medical Administration, National Health Commission of the People's Republic of China, Beijing, China; ^3^Department of Epidemiology and Biostatistics, Institute of Basic Medicine Sciences, Chinese Academy of Medical Sciences (CAMS), School of Basic Medicine, Peking Union Medical College, Beijing, China

**Keywords:** deep vein thrombosis (DVT), pharmacological prophylaxis, mechanical prophylaxis, septic shock, intestinal perforation

## Abstract

**Introduction:**

Septic shock, largely caused by intestinal perforation, is the most common critical illness in intensive care unit (ICU). As an important quality control strategy in ICU, deep vein thrombosis (DVT) prevention is routinely used in the treatment of septic shock. Nevertheless, the effects of DVT prevention on septic shock are not fully revealed. This study was thus designed to investigate the effects of DVT prevention on septic shock caused by intestinal perforation in China.

**Methods:**

A total of 463 hospitals were enrolled in a survey, led by the China National Critical Care Quality Control Center (China NCCQC) from January 1, 2018 to December 31, 2018. The association between DVT prevention, including pharmacological prophylaxis and mechanical prophylaxis, and outcomes, such as prognosis, complications, hospital stays, and hospitalization costs, was determined in the present study.

**Main Results:**

Notably, the increased rates of DVT prevention were not associated with the onset of complications in patients with septic shock caused by intestinal perforation (*p* > 0.05). In addition, even though increased DVT prevention did not affect hospital stays, it significantly decreased the discharge rates without doctor's order in patients with septic shock caused by intestinal perforation (*p* < 0.05). Nevertheless, it should be noted that the rates of pharmacological prophylaxis but not mechanical prophylaxis were significantly associated with the costs of septic shock caused by intestinal perforation (*p* < 0.05). Although increased total rates of DVT prevention and the rates of mechanical prophylaxis did not reduce the mortality in patients with septic shock caused by intestinal perforation, the higher frequent intervention using pharmacological prophylaxis indicated the lower mortality of these patients (*p* < 0.05).

**Conclusions:**

DVT prevention by any means is a safe therapeutic strategy for treating septic shock caused by intestinal perforation, and pharmacological prophylaxis reduced the mortality of patients with septic shock caused by intestinal perforation.

## Introduction

Septic shock is the most common critical illness in intensive care unit (ICU), with long hospital stay, high cost, and high mortality (1–3). Intestinal perforation is one of the important causes of septic shock (4, 5). Sepsis leads to complications in coagulation patients (6). Severe sepsis predisposes patients toward deep vein thrombosis (DVT) due to risk factors. Thromboembolic complications are observed in approximately one out of every 32 severe sepsis patients in India (7). The incidence of proximal DVT among medical patients hospitalized for at least two days was 2% for the year 2006 in China (8). Loss of DVT can lead to pulmonary embolism, hypoxemia and even death (9). DVT prevention rate is an important quality control index in ICU (10). DVT prophylaxis methods include pharmacological prophylaxis and mechanical prophylaxis (11). There are few reports on effects of DVT prevention on patients with septic shock caused by intestinal perforation. The purpose of this study is to investigate effects of DVT prevention on septic shock caused by intestinal perforation and to find a new way for the treatment of such patients.

## Methods

### Study Design

In this survey, 463 hospitals were enrolled in the Quality Improvement of Critical Care Program (QICC Program), led by the China National Critical Care Quality Control Center (China-NCCQC). These hospitals admitted 10,310 septic shock patients with intestinal perforation. The data were collected between January 1, 2018 and December 31, 2018. Informed consent was obtained from every study participating hospital. All information of participating hospitals and patients were list in Supplementary Tables 1, 2.

In this study, prevention methods of DVT include pharmacological prophylaxis and mechanical prophylaxis. The outcomes included hospital stays, hospitalization costs, complications, and prognosis. The complications included acute respiratory distress syndrome (ARDS), acute kidney injury (AKI), and acute liver failure (ALF). Based on data obtained from this survey, we will analyze effects of different DVT prevention methods on septic shock caused by intestinal perforation in China.

The authors are accountable for all aspects of the work in ensuring that questions related to the accuracy or integrity of any part of the work are appropriately investigated and resolved. The study was conducted in accordance with the Declaration of Helsinki (as revised in 2013). The central institutional review board approved the trial protocol at Peking Union Medical College Hospital (No. S-K1297), and individual consent for this retrospective analysis was waived.

The datasets supporting the conclusions of this article are included within the article and its additional files.

### Data Analysis

Statistical analysis was performed using SPSS software, version 16.0 (Chicago, IL, USA). All of the data are compared using trend test. All of the statistical tests were two-tailed, and a *p* < 0.05 was considered to be statistically significant.

## Results

### Relationship Between DVT Prevention and Complications

The increased rates of DVT prevention by any means were independent of complications in patients with septic shock caused by intestinal perforation (Table 1).

**Table 1:** Relationship between deep vein thrombosis (DVT) prevention and complications.

**Rates (n%)**	**ARDS**	***P*** **for trend**	**AKI**	***P*** **for trend**	**ALF**	***P*** **for trend**
DVT prevention		0.78		0.02		0.02
<80	142 (2.69)		548 (10.38)		68 (1.29)	
80~	8 (1.20)		64 (9.64)		2 (0.30)	
85~	22 (2.28)		104 (10.80)		16 (1.66)	
90~	27 (3.15)		71 (8.28)		8 (0.93)	
95~	68 (2.67)		225 (8.84)		16 (0.63)	
Rates of pharmacological prophylaxis		0.32		0.62		0.39
<20	88 (2.47)		321 (9.02)		39 (1.10)	
20~	42 (2.49)		182 (10.78)		20 (1.18)	
30~	13 (1.00)		131 (10.08)		13 (1.00)	
40~	66 (5.92)		143 (12.84)		18 (1.62)	
50~	58 (2.19)		235 (8.87)		20 (0.76)	
Rates of mechanical prophylaxis		0.48		0.06		0.19
<30	70 (2.41)		306 (10.53)		41 (1.41)	
30~	59 (3.17)		168 (9.03)		16 (0.86)	
50~	59 (3.01)		225 (11.49)		21 (1.07)	
70~	43 (2.03)		184 (8.67)		22 (1.04)	
90~	36 (2.46)		129 (8.81)		10 (0.68)	

*ARDS, acute respiratory distress syndrome; AKI, acute kidney injury; ALF, acute liver failure*.

### Relationship Between DVT Prevention and Hospital Stay

The increased rates of DVT prevention by any means were independent of hospital stays in patients with septic shock caused by intestinal perforation. Nearly half of the hospitals had <80% DVT prevention rates. The rates of pharmacological prophylaxis were <20% in a third of the hospitals. Most patients were hospitalized for <20 days (Table 2).

**Table 2:** Relationship between deep vein thrombosis (DVT) prevention and hospital stay.

**Hospital stay (day)**	**1–10**	**11–20**	**21–30**	**≥31**	***P*** **for trend**
Rates of DVT prevention (n%)					0.93
<80	1,852 (35.07)	1,893 (35.85)	821 (15.55)	715 (13.54)	
80~	256 (38.55)	235 (35.39)	82 (12.35)	91 (13.70)	
85~	359 (37.28)	319 (33.13)	145 (15.06)	140 (14.54)	
90~	289 (33.68)	292 (34.03)	156 (18.18)	121 (14.10)	
95~	914 (35.93)	898 (35.30)	397 (15.61)	335 (13.17)	
Rates of pharmacological prophylaxis (n%)					0.57
<20	1,256 (35.28)	1,268 (35.62)	556 (15.62)	480 (13.48)	
20~	627 (37.14)	607 (35.96)	252 (14.93)	202 (11.97)	
30~	473 (36.38)	480 (36.92)	170 (13.08)	177 (13.62)	
40~	401 (36.00)	381 (34.20)	177 (15.89)	155 (13.91)	
50~	913 (34.48)	901 (34.03)	446 (16.84)	388 (14.65)	
Rates of mechanical prophylaxis (n%)					0.60
<30	972 (33.46)	1,050 (36.14)	486 (16.73)	397 (13.67)	
30~	693 (37.26)	654 (35.16)	271 (14.57)	242 (13.01)	
50~	727 (37.13)	673 (34.37)	288 (14.71)	270 (13.79)	
70~	773 (36.41)	741 (34.90)	330 (15.54)	279 (13.14)	
90~	505 (34.49)	519 (35.45)	226 (15.44)	214 (14.62)	

### Relationship Between DVT Prevention and Hospitalization Costs

The total rates of DVT prevention and the rates of mechanical prophylaxis were not associated with the costs of septic shock caused by intestinal perforation. However, increased rates of pharmacological prophylaxis increased costs in patients with septic shock caused by intestinal perforation (*p* < 0.05) (Table 3).

**Table 3:** Relationship between deep vein thrombosis (DVT) prevention and hospitalization costs.

**Costs (RMB) (thousand yuan)**	** <30**	**30–59.9**	**60–89.9**	**≥90**	***P*** **for trend**
Rates of DVT prevention (n%)					0.30
<80	1,367 (25.89)	1,671 (31.64)	896 (16.97)	1,347 (25.51)	
80~	186 (28.01)	187 (28.16)	117 (17.62)	174 (26.20)	
85~	172 (17.86)	277 (28.76)	186 (19.31)	328 (34.06)	
90~	233 (27.16)	266 (31.00)	160 (18.65)	199 (23.19)	
95~	656 (25.79)	862 (33.88)	438 (17.22)	588 (23.11)	
Rates of pharmacological prophylaxis (n%)					<0.01
<20	984 (27.64)	1,157 (32.5)	574 (16.12)	845 (23.74)	
20~	451 (26.72)	520 (30.81)	302 (17.89)	415 (24.59)	
30~	269 (20.69)	426 (32.77)	233 (17.92)	372 (28.62)	
40~	236 (21.18)	359 (32.23)	190 (17.06)	329 (29.53)	
50~	674 (25.45)	801 (30.25)	498 (18.81)	675 (25.49)	
Rates of mechanical prophylaxis (n%)					0.91
<30	763 (26.27)	930 (32.01)	490 (16.87)	722 (24.85)	
30~	458 (24.62)	595 (31.99)	353 (18.98)	454 (24.41)	
50~	474 (24.21)	584 (29.83)	336 (17.16)	564 (28.80)	
70~	518 (24.40)	660 (31.09)	384 (18.09)	561 (26.42)	
90~	401 (27.39)	494 (33.74)	234 (15.98)	335 (22.88)	

### Relationship Between DVT Prevention and Prognosis

The improvement of the total rates of DVT prevention and the rates of mechanical prophylaxis increased the discharge rates under doctor's order (*p* < 0.05), decreased the discharge rates without doctor's order (*p* < 0.05), and was not associated with mortality. The increased rates of pharmacological prophylaxis increased the discharge rates under doctor's order, decreased the discharge rates without doctor's order, and reduced mortality of patients with septic shock caused by intestinal perforation (*p* < 0.05) (Table 4).

**Table 4:** Relationship between deep vein thrombosis (DVT) prevention and prognosis.

**Rates (n%)**	**Discharge under doctor's order**	***P*** **for trend**	**Discharge without doctor's order**	***P*** **for trend**	**Death**	***P*** **for trend**
DVT prevention		<0.01		0.04		0.53
<80	3,164 (59.91)		1,150 (21.78)		615 (11.65)	
80~	424 (63.86)		107 (16.11)		90 (13.55)	
85~	583 (60.54)		182 (18.90)		149 (15.47)	
90~	551 (64.22)		150 (17.48)		119 (13.87)	
95~	1,623 (63.8)		517 (20.32)		294 (11.56)	
Pharmacological prophylaxis		<0.01		<0.01		0.01
<20	2,122 (59.61)		797 (22.39)		442 (12.42)	
20~	1,018 (60.31)		354 (20.97)		189 (11.20)	
30~	805 (61.92)		242 (18.62)		197 (15.15)	
40~	720 (64.63)		182 (16.34)		128 (11.49)	
50~	1,680 (63.44)		531 (20.05)		311 (11.74)	
Mechanical prophylaxis		<0.01		<0.01		0.70
<30	1,832 (63.06)		612 (21.07)		313 (10.77)	
30~	1,010 (54.30)		435 (23.39)		290 (15.59)	
50~	1,212 (61.90)		370 (18.90)		244 (12.46)	
70~	1,325 (62.41)		416 (19.59)		264 (12.44)	
90~	966 (65.98)		273 (18.65)		156 (10.66)	

## Discussion

This study found that the increased rates of DVT prevention were not associated with the onset of complications in patients with septic shock caused by intestinal perforation. In addition, even though increased DVT prevention did not affect hospital stays, it significantly decreased the discharge rates without doctor's order in patients with septic shock caused by intestinal perforation. Nevertheless, it should be noted that the rates of pharmacological prophylaxis but not mechanical prophylaxis were significantly associated with the costs of septic shock caused by intestinal perforation. Although increased total rates of DVT prevention and the rates of mechanical prophylaxis did not reduce the mortality in patients with septic shock caused by intestinal perforation, the higher frequent intervention using pharmacological prophylaxis indicated the lower mortality of these patients.

The coagulation disorder of sepsis manifests from mild alterations to disseminated intravascular coagulation (12). First, the coagulation system is activated through the effect of chemical mediators on endothelium and monocytes in sepsis. Second, pro-inflammatory cascade leads to indirect coagulation. Sepsis predisposes patients toward DVT due to risk factors such as advanced age, chronic cardiopulmonary disease, recent surgery, immobilization, in-dwelling vascular catheters, and previous DVT history (13). The international guidelines recommend DVT prophylaxis with low-dose unfractionated heparin or daily low molecular weight heparin (LMWH) in patients with severe sepsis unless contraindicated. Moreover, patients with a contraindication for heparin should receive mechanical prophylactic devices (14). Nevertheless, the safety and effectiveness of DVT prevention on septic shock caused by intestinal perforation are not fully revealed. So we design this study to investigate the effects of DVT prevention on septic shock caused by intestinal perforation in China.

In theory, DVT causes pulmonary embolism, which causes ARDS. Therefore the increased rates of DVT prevention should reduce the occurrence of ARDS. However, due to the low proportion of severe pulmonary embolism caused by DVT (15), the increased rates of DVT prevention was not associated with the occurrence of ARDS in patients with septic shock caused by intestinal perforation in our research. One study found that the use of pharmacomechanical thrombolysis for treatment of acute DVT conferred a risk of AKI that will progress to chronic renal failure in a small fraction of affected patients (16). Another study found that patients prescribed ≥28 days of postoperative DVT prophylaxis exhibited significantly lower rates of AKI as compared to those prescribed short-duration prophylaxis (17). In our study, the increased rate of DVT prevention was not associated with the occurrence of acute kidney injury and acute liver failure in patients with septic shock caused by intestinal perforation. The differences in the results of these studies may be due to the different populations they included. Compared with common postoperative patients, there are too many factors affecting the occurrence of acute kidney injury and acute liver failure in patients with septic shock. In a word, our study showed that DVT prevention by any means is a safe therapeutic strategy for treating septic shock caused by intestinal perforation, and the increased rates of DVT prevention were not associated with the onset of complications.

Hospital stay is an important index to measure the therapeutic effect of sepsis and septic shock. This survey found that increased DVT prevention did not change hospital stays in patients with septic shock caused by intestinal perforation. There are several reasons for above result. On the one hand, DVT itself will not directly affect the length of hospital stay (18); on the other hand, there are many factors affecting the length of hospital stay in patients with septic shock caused by intestinal perforation (19–21). One single intervention may not change the hospital stays eventually.

From the foregoing, DVT prevention was not associated with the hospital stays in patients with septic shock caused by intestinal perforation. At the same time, mechanical prophylaxis itself is free or only a small charge in China (22), so increased mechanical prophylaxis did not affect the costs of patients with septic shock caused by intestinal perforation in this survey. Unlike mechanical prophylaxis, drugs to prevent DVT, such as low-molecular-weight heparin, are expensive (23), so increased pharmacological prophylaxis naturally lead to increased costs for patients with septic shock caused by intestinal perforation in China. How to control and even reduce the cost of DVT prevention is a subject that needs to be studied in future.

The increased rates of DVT prevention reduces the risk of pulmonary embolism in theory, leading to a reduction in expected mortality. Lower expected mortality lead to a reduction in the discharge rates without doctor's order. We observed that increased DVT prevention significantly decreased the discharge rates without doctor's order in patients with septic shock caused by intestinal perforation. The observation is in agreement with the theoretical hypothesis. However, in our study, increased total rates of DVT prevention and the rates of mechanical prophylaxis did not reduce the mortality. The reasons for the above results may be the incidence of fatal pulmonary embolism caused by DVT itself was extremely low (15), mechanical prophylaxis alone may not associated with mortality. In contrast to mechanical prophylaxis, drugs for DVT prevention, such as heparin and other drugs, not only reduce the occurrence of DVT, but also change the hemodynamic state in general (24). In addition, such drugs have anti-inflammatory and vascular endothelial protection effects (25–27). As a result, pharmacological prophylaxis eventually reduced the mortality in patients with septic shock caused by intestinal perforation in our research. Our results further support the international guidelines recommendation for pharmacological prophylaxis in patients with severe sepsis unless contraindicated.

There are some limitations of our study. First, factors such as compliance with the saving sepsis campaign guidelines can affect the efficacy and prognosis of sepsis treatment, and there is a high degree of consistency between the completion of DVT prevention and compliance with the saving sepsis campaign guidelines. This retrospective study cannot exclude the bias caused by these factors. More perfect prospective experiments should be designed to show the independent effect of DVT intervention by adjusting other interventions in the future. Second, since only one year's data was included in this study, the effects of DVT prevention on septic shock caused by intestinal perforation could not be analyzed continuously and dynamically. Third, those hospitals enrolled from China-NCCQC might have greater access to improve rates of DVT prevention by any means than other hospitals. Further studies will be necessary to determine the effects of DVT prevention on septic shock caused by intestinal perforation in China that differ in characteristics from those that participated.

## Conclusions

DVT prevention by any means is a safe therapeutic strategy for treating septic shock caused by intestinal perforation. Increased DVT prevention significantly decreased the discharge rates without doctor's order in patients with septic shock caused by intestinal perforation. Pharmacological prophylaxis reduced the mortality of patients with septic shock caused by intestinal perforation.

## Data Availability Statement

The original contributions presented in the study are included in the article/Supplementary Material, further inquiries can be directed to the corresponding author/s.

## Ethics Statement

The trial protocol was approved by the Central Institutional Review Board at Peking Union Medical College Hospital (NO: S-K1297). Written informed consent for participation was not required for this study in accordance with the national legislation and the institutional requirements. Written informed consent was obtained from the individual(s) for the publication of any potentially identifiable images or data included in this article.

## Author Contributions

XZ and DL: conception and design. YG and YL: administrative support. LW, XM, HH, and LS: provision of study materials or patients. LW, XM, HH, LS, YG, GS, YW, XZ, DL, and YL: collection, assembly of data, and manuscript writing. GS and YW: data analysis and interpretation. All authors contributed to the article and approved the submitted version.

## Funding

This work was supported by National Natural Science Foundation of China (No. 81801901), National Key R&D Program of China (No. 2020YFC0861000) and China Medical Board (No. CMB 20-381). The funders had no role in the study's design and conduct; collection, management, analysis, and interpretation of the data; preparation, review, or approval of the manuscript; and decision to submit the manuscript for publication.

## Conflict of Interest

The authors declare that the research was conducted in the absence of any commercial or financial relationships that could be construed as a potential conflict of interest.

## Publisher's Note

All claims expressed in this article are solely those of the authors and do not necessarily represent those of their affiliated organizations, or those of the publisher, the editors and the reviewers. Any product that may be evaluated in this article, or claim that may be made by its manufacturer, is not guaranteed or endorsed by the publisher.

## Abbreviations

ICU: intensive care unit
DVT: deep vein thrombosis
QICC Program: Quality Improvement of Critical Care Program
China-NCCQC: China National Critical Care Quality Control Center
ARDS: acute respiratory distress syndrome
AKI: acute kidney injury
ALF: acute liver failure.

## References

[1] Goodman S Sprung CL Ziegler D Weiss YG. Cortisol changes among patients with septic shock and the relationship to ICU and hospital stay. Intensive Care Med. (2005) 31:1362–9. 10.1007/s00134-005-2770-6 16151722

[2] Dupuis C Bouadma L Ruckly S Perozziello A Van-Gysel D Mageau A. Sepsis and septic shock in France: incidences, outcomes and costs of care. Ann Intensive Care. (2020) 10:145. 10.1186/s13613-020-00760-x 33079281 PMC7575668

[3] Kozyrakis D Kratiras Z Soukias G Chatzistamou SE Zarkadas A Perikleous S. Clinical outcome and prognostic factors of sepsis, septic shock and prolonged hospitalization, of patients presented with acute obstructive pyelonephritis. J Endourol. (2020) 34:516–22. 10.1089/end.2019.0801 32000528

[4] Notaro S Sorrentino M Ruocco A Notaro A Corcione A Murino P. Combined use of high doses of vasopressin and corticosteroids in a patient with Crohn's disease with refractory septic shock after intestinal perforation: a case report. J Med Case Rep. (2017) 11:320. 10.1186/s13256-017-1456-3 29129185 PMC5682640

[5] Abdalaziz FA Algebaly HAF Ismail RI El-Sherbini SA Behairy A. The use of bedside echocardiography for measuring cardiac index and systemic vascular resistance in pediatric patients with septic shock. Revista Brasileira de terapia intensiva. (2018) 30:460–70. 10.5935/0103-507X.20180067 30672970 PMC6334480

[6] Boddi M Peris A. Deep vein thrombosis in intensive care. Adv Exp Med Biol. (2017) 906:167–81. 10.1007/5584_2016_114 27628009

[7] Levi M Levy M Williams MD Douglas I Artigas A Antonelli M. Prophylactic heparin in patients with severe sepsis treated with drotrecogin alfa (activated). Am J Respir Crit Care Med. (2007) 176:483–90. 10.1164/rccm.200612-1803OC 17556722

[8] Cheng G Chan C Liu YT Choy YF Wong MM Yeung PK. Incidence of deep vein thrombosis in hospitalized Chinese medical patients and the impact of DVT prophylaxis. Thrombosis. (2011) 2011:629383. 10.1155/2011/629383 22084666 PMC3211080

[9] Waheed SM Kudaravalli P Hotwagner DT. Deep Vein Thrombosis. Treasure Island, FL: StatPearls. (2021).

[10] Kumar A Mehta Y Ali T Gupta MK George JV. Deep vein thrombosis in medical and surgical Intensive Care Unit patients in a Tertiary Care Centre in North India: Incidence and risk factors. J Anaesthesiol Clin Pharmacol. (2017) 33:181–6. 10.4103/0970-9185.209760 28781442 PMC5520589

[11] Badireddy M Mudipalli VR. Deep Venous Thrombosis Prophylaxis. Treasure Island, FL: StatPearls. (2021). 30521286

[12] Saracco P Vitale P Scolfaro C Pollio B Pagliarino M Timeus F. The coagulopathy in sepsis: significance and implications for treatment. Pediatr Rep. (2011) 3:e30. 10.4081/pr.2011.e30 22355515 PMC3283198

[13] Dellinger RP. Inflammation and coagulation: implications for the septic patient. Clin Infect Dis. (2003) 36:1259–65. 10.1086/374835 12746771

[14] Evans L Rhodes A Alhazzani W Antonelli M Coopersmith CM French C. Surviving sepsis campaign: international guidelines for management of sepsis and septic shock 2021. Intensive Care Med. (2021) 47:1181–247. 10.1007/s00134-021-06506-y 34599691 PMC8486643

[15] Stubbs MJ Mouyis M Thomas M. Deep vein thrombosis. BMJ. (2018) 360:k351. 10.1136/bmj.k351 29472180

[16] Salem KM Saadeddin Z Go C Malak OA Eslami MH Hager E. Risk factors for acute kidney injury after pharmacomechanical thrombolysis for acute deep vein thrombosis. J Vasc Surg Venous Lymphat Disord. (2021) 9:868–73. 10.1016/j.jvsv.2020.11.005 33186753 PMC8107181

[17] Durand WM Goodman AD Johnson JP Daniels AH. Assessment of 30-day mortality and complication rates associated with extended deep vein thrombosis prophylaxis following hip fracture surgery. Injury. (2018) 49:1141–8. 10.1016/j.injury.2018.03.019 29580646

[18] Di Nisio M van Es N Buller HR. Deep vein thrombosis and pulmonary embolism. Lancet. (2016) 388:3060–73. 10.1016/S0140-6736(16)30514-1 27375038

[19] Daniels LM Durani U Barreto JN O'Horo JC Siddiqui MA Park JG. Impact of time to antibiotic on hospital stay, intensive care unit admission, and mortality in febrile neutropenia. Support Care Cancer. (2019) 27:4171–7. 10.1007/s00520-019-04701-8 30805726

[20] Fernandez-Sarmiento J Carcillo JA Salinas CM Galvis EF Lopez PA Jagua-Gualdron A. Effect of a sepsis educational intervention on hospital stay. Pediatr Crit Care Med. (2018) 19:e321–8. 10.1097/PCC.0000000000001536 29543684

[21] Rodriguez-Homs LG Masoud SJ Mosca MJ Jawitz OK O'Brien C Mosca PJ. Greater compliance with early sepsis management is associated with safer care and shorter hospital stay. J Healthc Qual. (2021) 43:347–54. 10.1097/JHQ.0000000000000295 34734919

[22] Zhao W McArthur A Yu Z Hu Y Luo J. Prevention of venous thromboembolism in postoperative abdominal patients: a best practice implementation project. JBI Database System Rev Implement Rep. (2018) 16:1887–901. 10.11124/JBISRIR-2017-003665 30204673

[23] Yang L Wu J. Cost-effectiveness of rivaroxaban compared with enoxaparin plus warfarin for the treatment of hospitalised acute deep vein thrombosis in China. BMJ Open. (2020) 10:e038433. 10.1136/bmjopen-2020-038433 32737096 PMC7394175

[24] Weiss RJ Esko JD Tor Y. Targeting heparin and heparan sulfate protein interactions. Org Biomol Chem. (2017) 15:5656–68. 10.1039/C7OB01058C 28653068 PMC5567684

[25] Hao C Xu H Yu L Zhang L. Heparin: an essential drug for modern medicine. Prog Mol Biol Transl Sci. (2019) 163:1–19. 10.1016/bs.pmbts.2019.02.002 31030744

[26] Abbadi A Loftis J Wang A Yu M Wang Y Shakya S. Heparin inhibits proinflammatory and promotes anti-inflammatory macrophage polarization under hyperglycemic stress. J Biol Chem. (2020) 295:4849–57. 10.1074/jbc.RA119.012419 32107314 PMC7152777

[27] Camprubi-Rimblas M Tantinya N Guillamat-Prats R Bringue J Puig F Gomez MN. Effects of nebulized antithrombin and heparin on inflammatory and coagulation alterations in an acute lung injury model in rats. J Thromb Haemost. (2020) 18:571–83. 10.1111/jth.14685 31755229 PMC9906372

