# Supplementary material for: Association Between Different DVT Prevention Methods and Outcomes of Septic Shock Caused by Intestinal Perforation in China: A Cross-Sectional Study
Source: Front Med (Lausanne). 2022 Apr 27;9:878075. doi: 10.3389/fmed.2022.878075 (PMC9092133; doi:10.3389/fmed.2022.878075)
Supplement: Supplementary file 1 [file Table_1.DOCX]

**Supply table 1. Basic information of patients**

|  | N | n% |
| --- | --- | --- |
| total | 10310 | 100 |
| gender |  |  |
| male | 6809 | 66.04 |
| female | 3501 | 33.96 |
| hospital stay (day) |  |  |
| 1-10 | 3670 | 35.60 |
| 11-20 | 3637 | 35.28 |
| 21-30 | 1601 | 15.53 |
| ≥31 | 1402 | 13.60 |
| costs (RMB) (thousand yuan) |  |  |
| <30 | 2614 | 25.35 |
| 30-59.9 | 3263 | 31.65 |
| 60-89.9 | 1797 | 17.43 |
| ≥90 | 2636 | 25.57 |
| way of discharge |  |  |
| discharge under doctor's order | 6345 | 61.54 |
| discharge without doctor's order | 2106 | 20.43 |
| death | 1267 | 12.29 |
