# Supplementary material for: Association Between Different DVT Prevention Methods and Outcomes of Septic Shock Caused by Intestinal Perforation in China: A Cross-Sectional Study
Source: Front Med (Lausanne). 2022 Apr 27;9:878075. doi: 10.3389/fmed.2022.878075 (PMC9092133; doi:10.3389/fmed.2022.878075)
Supplement: Supplementary file 2 [file Table_2.DOCX]

**Supply table 2. Information of deep vein thrombosis (DVT) prevention**

| Rates (n%) | number of hospital | number of patients |
| --- | --- | --- |
| total | 463 | 10310 |
| DVT prevention |  |  |
| <80 | 207(44.71) | 5281(51.22) |
| 80~ | 32(6.91) | 664(6.44) |
| 85~ | 41(8.86) | 963(9.34) |
| 90~ | 53(11.45) | 858(8.32) |
| 95~ | 130(28.08) | 2544(24.68) |
| pharmacological prophylaxis |  |  |
| <20 | 144(31.1) | 3560(34.53) |
| 20~ | 82(17.71) | 1688(16.37) |
| 30~ | 60(12.96) | 1300(12.61) |
| 40~ | 47(10.15) | 1114(10.81) |
| 50~ | 130(28.08) | 2648(25.68) |
| mechanical prophylaxis |  |  |
| <30 | 107(23.11) | 2905(28.18) |
| 30~ | 75(16.2) | 1860(18.04) |
| 50~ | 82(17.71) | 1958(18.99) |
| 70~ | 113(24.41) | 2123(20.59) |
| 90~ | 86(18.57) | 1464(14.20) |
